# The Second International Symposium on Languages in Biology and Medicine (LBM) 2007

**DOI:** 10.1186/1471-2105-9-S3-S1

**Published:** 2008-04-11

**Authors:** Su Jian, Christopher JO Baker

**Affiliations:** 1 LBM 2007 PC Chairs; 2 Data Mining Department, Institute for Infocomm Research, 21 Heng Mui Keng Terrace, Singapore 119613

The 2nd International Symposium on Languages in Biology and Medicine (LBM2007) was held in Singapore in December 2007. It provided a renewed opportunity for interaction between language professionals with different methodological backgrounds. In particular original research and applications of language technologies in biology and medicine were solicited. The relevant themes are listed below.

· Natural language: text mining, retrieval and management;

· Ontology language: ontology construction, extension and management;

· Logic language: knowledge representation and induction;

· Sequence language: RNA structure prediction, protein domain prediction;

· Database language: database interface, query language;

· Visualization language: information visualization, molecular visualization;

A total of 47 submissions were received and reviewed by a 37 strong program committee and 19 additional reviewers from Asia, Europe and North America. The committee represented the six afore mentioned research disciplines and participated in a double blind review process with 3 reviews per paper. A selection of 12 papers was accepted (25.5% acceptance rate) for long oral presentations during the symposium and publication in the LBM special issue of BMC Bioinformatics. A further 11 out of the remaining 35 papers were selected for short oral presentation (31.4% acceptance rate).

This LBM special issue of *BMC Bioinformatics* consists of 10 long oral presentation papers, as some papers were withdrawn due to unforeseen circumstances. A proceedings of LBM short paper presentations comprising of 7 papers was published by CEUR and is available at . From these two editions 8 papers are from Asia (China 1, Japan 3, Korea 1, Singapore 1, TaiWan 2), and 9 papers are from Europe and North America (Finland 1, Hungary 1, Sweden 1, UK 4, Canada 1, USA 1). These papers were presented in five sessions, namely (a) Terminology and Named Entities, (b) Text Classification 1, (c) Text Classification 2, (d) Text Mining, (e) Ontology and Logic.

In addition to technical paper presentations, the 3 keynote presentations of the symposium addressed terminology integration (Olivier Bodenreider), text mining services (Sophia Ananiadou) and ontology alignment (Patrick Lambrix). The panel discussion chaired by Junichi Tsujii concluded the symposium with an examination of the synergies among the biomedical language and knowledge technologies.

In conclusion we express our deep appreciation to the program committee members and the additional reviewers who worked on a very tight schedule, sharing their valuable time and formidable expertise in support of the LBM review process. We also thank, Ho-Joon Lee from KAIST and Chen Bin from I2R / NUS for their assistance with the EasyChair system, the LBM website and other miscellaneous tasks. We also wish to thank Jong C. Park, Limsoon Wong, the two general chairs and See Kiong Ng for their help and suggestions.

